# Baseline Characteristics of Bronchial Secretions and Bronchoalveolar Lavage Fluid in Patients with Ventilator-Associated Pneumonia

**DOI:** 10.3390/microorganisms13030676

**Published:** 2025-03-18

**Authors:** Rodopi Stamatiou, Efrosyni Gerovasileiou, Maria Angeli, Konstantina Deskata, Vasiliki Tsolaki, Konstantinos Mantzarlis, Epameinondas Zakynthinos, Demosthenes Makris

**Affiliations:** 1 Intensive Care Unit, University Hospital of Larissa, Faculty of Medicine, School of Health Sciences, University of Thessaly, BIOPOLIS, 41500 Larissa, Greecedimomakris@uth.gr (D.M.); 2 Department of Biochemistry and Biotechnology, School of Health Sciences, University of Thessaly, BIOPOLIS, 41500 Larissa, Greece

**Keywords:** multidrug-resistant infections, ventilator-associated pneumonia, mechanical ventilation, biomarkers, mechanically ventilated

## Abstract

Mechanically ventilated (MV) patients often develop ventilator-associated pneumonia (VAP) with increased mortality risk, especially in VAP caused by multidrug-resistant (MDR) microorganisms. We evaluated MV patients and monitored VAP presentation, microbiologically confirmed. The patients underwent bronchoalveolar lavage (BAL) and blind bronchial aspiration (AC) at baseline. Systematic bronchial secretion and radiologic assessments were performed daily. The patients were classified as MDR-VAP, non-MDR-VAP, or non-VAP. The APACHE II and SOFA scores, microbiology, inflammatory markers, respiratory system characteristics, and ventilator settings were evaluated. BAL and AC were assessed for total protein levels, cellular number and profile, and IL-1β and TNF-α levels. Of the VAP patients, 46.1% presented with MDR-VAP due to *Acinetobacter baumannii*, *Pseudomonas aeruginosa*, *Klebsiella pneumoniae*, or *Stenotrophomonas maltophilia*, and 53.8%—with non-MDR-VAP. The VAP patients had higher APACHE II scores and airway pressure but a lower baseline PO_2_/FIO_2_ compared to the non-VAP patients, while PO_2_/FIO_2_ was increased in MDR-VAP compared to non-MDR-VAP. BAL protein, IL-1β, and cellular levels were increased in VAP vs. non-VAP and in non-MDR-VAP compared to MDR-VAP. Macrophages and polymorphonuclears were 34.36% and 23.76% in VAP, statistically significant increased compared to non-VAP. Their percentages were also increased in non-MDR-VAP compared to MDR-VAP. These differences imply a different immunological profile in non-MDR-VAP patients. In conclusion, MDR-VAP patients may present significant differences in baseline clinical characteristics and molecular biomarkers, which may help in prompt diagnosis and an improved therapeutic approach.

## 1. Introduction

Mechanically ventilated (MV) patients often appear to have increased airway inflammation among other conditions that are present in such patients. The most commonly observed lung complications in MV patients are atelectasis [1], ventilator-induced lung injury [2], and pneumothorax [3]. However, airway inflammation present in these patients has not yet been attributed to lung complications that occur during their intensive care unit (ICU) hospitalization or to the intubation itself.

Ventilator-associated pneumonia (VAP) is an infection that happens in MV patients after at least 48 h of intubation, with a prevalence of 25–30% of patients [4]. VAP is classified into early- and late-onset. Early-onset VAP occurs after 48–96 h of ICU admission and is commonly due to antimicrobial-sensitive bacteria. Late-onset VAP occurs any time after 96 h of ICU admission and is most probably due to multidrug-resistant (MDR) pathogens. However, several studies have described high rates of VAP attributed to MDR pathogens infecting MV patients during hospitalization [5,6,7]. VAP increases morbidity and mortality up to 75% and raises hospitalization costs and the level of care required [8]. Particularly, VAP caused by MDR bacteria is related to a significant attributable mortality [5,6]. Many risk factors are associated with the occurrence of VAP due to MDR pathogens. Epidemiological data show that even though the prevalence rates of MDR bacteria are highly variable across different countries, the MDR-VAP diagnosis seems to have become more frequent recently [9]. Furthermore, previous antibiotic exposure is strongly associated with MDR involvement, while the immunosuppression state of the patients and the microbial profile in the hospital could also be major risk factors causing VAP attributed to MDR pathogens [8]. It is known that VAP is usually caused by bacterial infections, such as *Acinetobacter baumanii*, *Klebsiella pneumoniae*, and *Pseudomonas aeruginosa* [10]. These pathogens often colonize the oropharynx and the gut and are easily acquired through transmission by either healthcare specialists from various surfaces in the hospital or from other patients [11]. However, many species of these bacteria can be described as MDR pathogens [12,13]. Additionally, the clinical procedures that are performed during ICU stays, such as aspiration or intubation, can themselves further increase the possibility of VAP manifestation [14].

Early VAP diagnosis and characterization of the pathogen that caused VAP can lead to timely antibiotic treatment as well as the selection of an antibiotic that will be the most effective in fighting MDR bacteria. Even though VAP can be diagnosed using radiological and microbiological methods [15], a plethora of lung complications can lead to the clinical deterioration of MV patients. Furthermore, one-third of patients with VAP exhibit only the clinical criteria of sepsis [16], so when there is clinical suspicion of VAP, empirical antibiotic therapy should be immediately administered since the delayed administration of treatment is related to high rates of both morbidity and mortality [17,18]. Therefore, the need to establish reliable biomarkers of lung inflammation and VAP occurrence that are easy to measure and sufficiently reliable to be implemented in everyday risk evaluation of VAP manifestation is present.

There are studies that have proposed the use of protein and cell count evaluation in bronchial secretions to identify and estimate airway inflammation in MV patients [19,20]. Furthermore, the cellular profile in bronchoalveolar lavage (BAL) has been proposed as a biomarker for evaluating airway cellularity, since it can be prepared in a short time after sampling [20]. Additionally, there are studies showing that the percentage of different types of immune cells can be corelated to the induction of sepsis in MV patients [21]. The observations regarding the immunological profile of such patients can also be related to the duration of hospitalization prior to ICU admission as well as their overall clinical condition [22]. However, there have been questions raised regarding the need for a bronchoscopy in order to obtain a BAL sample, as well as about the clinical use of such a technique [23]. Therefore, the optimization of the sampling method, the type of samples to be examined, as well as the panel of biomarkers to be evaluated in such patients are of great interest. Additionally, the characterization of biomarkers that are indicative of possible MDR infections in MV patients could influence the therapeutic approach used. Furthermore, the identification of biomarkers that could assist in the prediction of which patients are more prone to VAP development as well as MDR infections is of great interest, since it could alter the therapeutic approach in such patients.

The aim of the present study was to examine whether the evaluation of clinical and molecular characteristics of MV patients could be indicative of possible VAP manifestation. Furthermore, the susceptibility to being infected by MDR pathogens was also assessed in these patients. As a secondary outcome, this study aimed to evaluate the most suitable sampling method that could produce biomaterials suitable for airway inflammation evaluation and prediction of VAP manifestation in MV patients. The panel of biomarkers that could be used to evaluate such parameters should be characterized as easy to use and fast monitoring biomarkers. The use of biomarkers’ evaluation at baseline, namely within the first 24 h of intubation in MV patients, aims to show the possible capability of such evaluations to characterize patients as high- or low-risk for VAP manifestation as early as possible. Such data could be helpful for therapeutic approach planning and the provision of more effective health care for critically ill patients.

## 2. Materials and Methods

### 2.1. Patients

This was a single-center prospective cohort study conducted in the ICU of a tertiary university hospital in Central Greece from 2022 to 2023. Twenty-nine patients were enrolled in the study. All of them fulfilled the following criteria: age >18 years old and mechanical ventilation >48 h. The exclusion criteria were purulent sputum or pneumonia on admission, new and persistent infiltrates on chest radiography within the first 48 h of ICU admission, diagnosed chronic obstructive pulmonary disease (COPD), and pregnancy. All the patients’ next of kin received information about the research protocol and the purpose of the study and then signed a consent form. Both the consent form and the research protocol were approved by the ethics committee of the university hospital where the study was conducted. All the data acquired from the patients’ files were anonymized and protected by code allocation. The approval of the Ethics Committee of the Medical Department and the University Hospital, School of Health Sciences, University of Thessaly, was obtained according to the Declaration of Helsinki and the STROBE guidelines (55366/23 December 2022). Patients were recruited if they underwent bronchoscopy in the first 24 h of their admission in the ICU. The patients were monitored for ventilator-associated pneumonia according to the international guidelines.

### 2.2. Outcomes

The primary outcome of the study was the baseline TNF-α BALF levels in the patients with VAP. Secondarily, we assessed the baseline TNF-α levels in the MDR-VAP and non-MDR-VAP patients and the IL-1 BALF levels in the aforementioned categories of patients.

The mean (SD) TNF-α BALF levels in MV patients in our previous study [19] were used for assessing the number of patients that would be required in each group (VAP and non-VAP) for a power of 91–100% (a = 0.05).

### 2.3. Microbial Evaluation

The identification and susceptibility testing of microorganisms in the VAP patients were carried out using a Vitek2 instrument (BioMérieux, Marcy l’Étoile, France). The categorization into MDR or non-MDR VAP was defined by an antibiogram in order to specify microbial resistance to antibiotics.

Antibiotic susceptibility testing was evaluated, and the specific antibiotic resistance profile of the isolated MDR pathogens was characterized by the microbiology laboratory at the University Hospital of Larissa according to the breakpoints of EUCAST issued in 2019 and CLSI (Clinical and Laboratory Standards Institute) as described in the 2020 performance standards for antimicrobial susceptibility testing.

### 2.4. Samples

Two different samples were acquired from the patients within 24 h of their intubation: a bronchoalveolar lavage (BAL) sample from the segmental airways with instillation of 120 mL of NaCl 0.9% during bronchoscopy (ΡΕΝΤΑΧ FΒ-15Χ, PENTAX Medical, PENTAX Europe GmbH, Hamburg, Germany) and a blind bronchial aspiration sample acquired by the attending nurse from the tracheal tube (AC). These sampling techniques were chosen due to the fact that BAL sampling is a commonly used technique for lung monitoring and provides clinicians with biomaterials useful for biomarker evaluation. Furthermore, AC samples have been found to be effective for biomarker evaluation as well and represent an easy-to-use noninvasive method of sampling [19]. Both samples were mechanically processed for mucus removal using a Heidolph Silent Crusher S (Heidolph Instruments GmbH & Co. KG, Schwabach, Germany) as previously described [19]. More specifically, 4 rounds of homogenization that lasted 20 s each, at maximum speed, with 15 s intervals, were used. All the samples were evaluated microscopically and macroscopically, and only the samples that were of good quality were used, namely if they did not have any contamination and if the percentage of epithelial cells was below 10%. Cell counts were performed by two independent observers using a hemocytometer after Trypan Blue staining for dead cell exclusion, while the estimation of the total protein and albumin levels was performed in the sample supernatant after centrifugation (1000× *g*, 20 min, 4 °C) of the mucus-dissolved sample.

#### 2.4.1. Evaluation of the Total Protein Levels

Total protein levels were estimated using the Bradford chromatometric method. This method is based on the absorbance that is detected in a photometer due to the binding of the Coomassie Brilliant Blue stain to protein molecules present in samples. A standard curve is used to define the quantity of proteins that is measured in each sample according to the absorbance measured [24].

#### 2.4.2. Evaluation of the Cytokine Levels

Both TNF-α and IL-1β levels were evaluated using the Human TNF-α ELISA Pro kit (Mabtech, Nacka Strand, Sweden) and the Human IL-1β ELISA Pro kit (Mabtech, Nacka Strand, Sweden) according to the manufacturer’s instructions. ELISA (enzyme-linked immunosorbent assay) is a method that uses a capture antibody specific for the antigen needed to be detected, coated onto a titer plate. This antibody binds to the antigen. The antigen also binds to a detection antibody that is added and then conjugated to a fluorophore antibody responsible for the recognition of the aforementioned binding. The color that is produced is measured using an ELISA reader. A standard curve with known antigen concentrations is used to assess the concentration of the antigen in the sample in question [25]. The material that was used for the evaluation of the cytokine levels was part of the same supernatant that was used for the evaluation of the total protein levels.

#### 2.4.3. Cell Count and Cell Type Evaluation

The cellular profile of the samples was evaluated after cytospin centrifugation and smear preparation using Giemsa–May–Grunwald staining and microscopic observations for cell type characterization. The type of cells was identified according to the staining profile (nuclear and cytoplasmic) that was present in the samples’ smears.

### 2.5. Statistical Analysis

All data are expressed as the means ± SEM, and N refers to the number of patients. The data were analyzed for normality with the D’Agostino–Pearson test. The descriptive statistics were assessed using one-way ANOVA when more than 2 groups were compared and the Mann–Whitney test when 2 groups with nonparametric values were compared. A comparison was considered significant with *p* < 0.05. The statistical analysis was performed using Graphpad Prism 5 Software, Boston, MA, USA.

## 3. Results

### 3.1. Clinical Characteristics in VAP Manifestations

Out of the 29 patients included in the study, 16 patients did not develop VAP, while of the 13 patients that were diagnosed with VAP, 7 were infected with non-MDR microorganisms (non-MDR-VAP), and 6 had MDR infections (MDR-VAP). Therefore, three groups of patients were included in the study: the non-VAP group, the MDR-VAP group, and the non-MDR-VAP group. The characteristics of the patients enrolled in the study are presented in Table 1 and Table 2. Namely, their mean age was 65.94 years old, their mean APACHE II score (Acute Physiology and Chronic Health Evaluation II) was 19.26, and their mean SOFA score (Sequential Organ Failure Assessment) was 8.7. The APACHE II score was significantly higher in the VAP group compared to the non-VAP group. Namely, the score was 22.46 ± 2.19 and 16.06 ± 2.05 in the VAP and non-VAP patients, respectively (* *p* < 0.05, Mann–Whitney test, Table 1). Furthermore, PO_2_/FiO_2_ was significantly lower in the VAP group, with a value of 180.5 ± 24.6 compared to the non-VAP group with a value of 274.4 ± 26.51 (** *p* < 0.01, Mann–Whitney test, Table 1). The pressure plateau measured on the admission day was higher in the VAP group compared to the non-VAP group, with the respective values of 24.33 ± 2.3 cm H_2_O for the VAP patients and 18.06 ± 0.95 cm H_2_O (** *p* < 0.01, Mann–Whitney test, Table 1).

A survival analysis using the baseline parameters such as ventilator settings (PEEP, Vt, P plateau, driving pressures), demographics, and clinical characteristics, including the antibiotics used, was performed but did not reveal any significant differences between survivors and non-survivors. We assume that this might have been due to either the small size of the population for such an analysis or because all the patients were ventilated using the same protocol of lung protective strategy and the same local protocol for antibiotics. Furthermore, we found no significant differences between the MDR-VAP and non-MDR-VAP patients with respect to demographics, including age, sex, comorbidities such as heart failure, asthma, COPD, APACHE score, and SOFA score on the admission day.

During the study period, MDR pathogens were isolated in 7 of the 13 VAP patients. At the time of admission, PO_2_/FiO_2_ was significantly lower in the MDR-VAP group compared to the non-MDR-VAP group. Namely, it was 153.6 ± 30.87 in MDR-VAP and 283.4 ± 26.96 in non-MDR-VAP (* *p* < 0.05, Mann–Whitney test, Table 2). A biochemical analysis of the blood samples received from the MV patients enrolled in the study showed that serum albumin levels were significantly lower in the patients who had MDR-VAP compared to the group of patients with non-MDR-VAP (*p < 0.05, Mann–Whitney test, Table 2).

### 3.2. Infection Characteristics

The data regarding VAP manifestation are presented in Table 3. The most common microorganisms that caused VAP were *Pseudomonas aeruginosa* and *Klebsiella pneumoniae*, while 2 cases of *Klebsiella* and 3 cases of *Pseudomonas* infections were resistant to all antibiotics (Table 3). The mean intubation time to the day of VAP manifestation was 7.85 vs. 5.16 days in the MDR-VAP and non-MDR-VAP patients, respectively (* *p* < 0.05, Mann–Whitney test, Table 3). In most cases, the lung was the source of sepsis for the patients. Antibiotic susceptibility was also assessed in the pathogens isolated from the MDR-VAP patients. More specifically, the percentages of resistance were as follows: meropenem, 71.43%; piperacillin/tazobactam, 85.71%; gentamicin, 57.14%; colistin, 57.14%; ampicillin, 100%; ampicillin/sulbactam, 85.71%; and ciprofloxacin, 100% (Table 4).

### 3.3. Total Protein Levels

When the total protein levels were evaluated in the bronchial secretions obtained with BAL and by blind aspiration (AC), they were increased in the BAL samples obtained from the patients suffering from VAP compared to the patients who were not diagnosed with VAP (* *p* < 0.05, one-way ANOVA, Table 5). Interestingly, no differences in albumin levels were observed in blood serum samples from the non-VAP patients compared to samples from the patients with VAP (Table 1). On the other hand, the total protein levels were elevated in the AC samples compared to the BAL samples obtained from the patients with non-MDR-VAP (* *p* < 0.05, one-way ANOVA, Table 4). Namely, the total protein levels were 7.86 ± 0.81 μg/mL in the BAL samples while the levels were 17 ± 1.85 μg/mL in the AC samples from the non-MDR-VAP patients (Table 4). However, no differences were observed in the protein levels between the MDR-VAP and non-MDR-VAP patients.

### 3.4. Cytokine Levels

Furthermore, only the IL-1β levels in the bronchial secretions were elevated in the VAP patients compared to the non-VAP patients (* *p* < 0.05, one-way ANOVA,) with the IL-1β levels being higher in the non-MDR-VAP patients compared to those infected with MDR microorganisms (* *p* < 0.05, one-way ANOVA, Table 4). Interestingly, the IL-1β levels were more elevated in the BAL samples compared to the AC samples (* *p* < 0.05, ** *p* < 0.01, one-way ANOVA, Table 4), while the opposite was observed regarding the TNF-α levels, which were higher in the AC samples compared to the BAL samples (* *p* < 0.05, ** *p* < 0.01, one-way ANOVA, Table 4) regardless of the type of infection, MDR or non-MDR, in the VAP patients.

### 3.5. Cell Count and Type Evaluation

**Figure 1:**
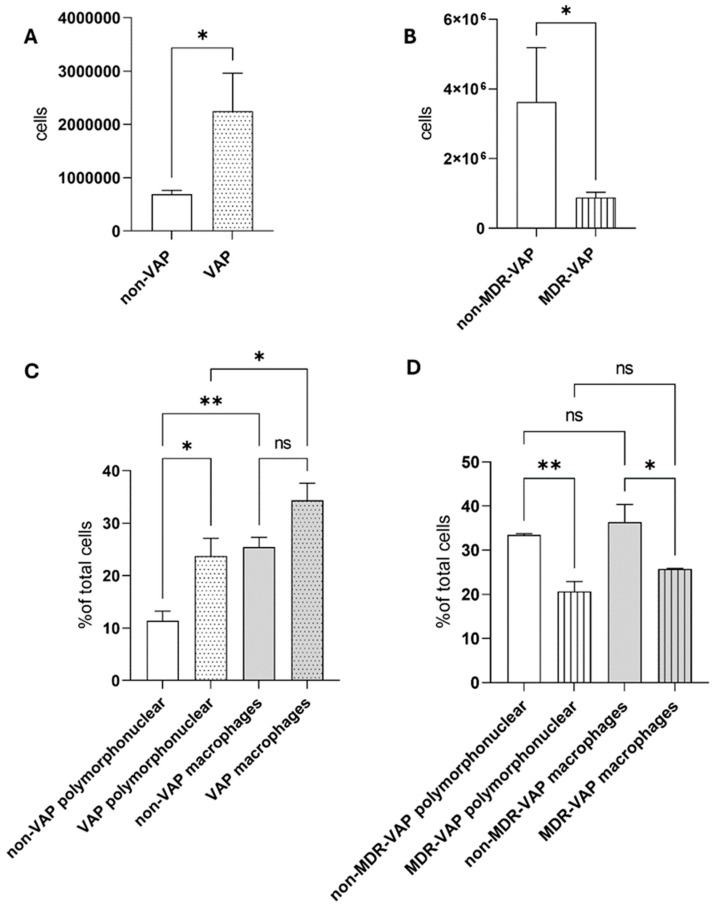
Cell count and cellular profile in the BAL samples from the MV patients. (**A**). Cell count was elevated in the BAL from the VAP patients. Note: * *p* < 0.05, Mann–Whitney test. (**B**). Cell count was elevated in the BAL samples from the non-MDR-VAP patients compared to the MDR-VAP patients. Note: * *p* < 0.05, Mann–Whitney test. (**C**). The number of polymorphonuclear cells was elevated in the VAP patients compared to the non-VAP patients, while the number of macrophages was higher than that of polymorphonuclear cells. Note: ns non-significant, * *p* < 0.05, ** *p* < 0.01, Mann–Whitney test compared to the relative group. (**D**). The non-MDR-VAP patients had elevated cell counts regardless of the type of cells. Note: ns non-significant, * *p* < 0.05, ** *p* < 0.01, Mann–Whitney test compared to the relative group.

As far as the total cell count is concerned, there were more cells counted in the BAL samples from the patients suffering from VAP than from the non-VAP patients (* *p* < 0.05, one-way ANOVA, Figure 1A). Namely, 687,981 ± 76,082 cells were counted in the BAL samples from the non-VAP patients compared to 2,398,295 ± 765,264 cells in same type of samples from the patients suffering from VAP (Figure 1A). Furthermore, the BAL samples from the VAP patients infected with non-MDR pathogens had higher cell counts compared to those who were suffering from MDR infections (* *p* < 0.05, one-way ANOVA, Figure 1B). Additionally, the cellular profile that was evaluated in the bronchial secretions from the patients suffering from VAP revealed an increase in polymorphonuclear cells, as well as in macrophages, compared to the non-VAP patients regardless of the sample type and/or the cell type (* *p* < 0.05, one-way ANOVA, Figure 1C). In addition to this observation, the same increase was found in the MDR-VAP patients compared to the non-MDR-VAP ones (* *p* < 0.05, ** *p* < 0.01, one-way ANOVA, Figure 1D). As far as the cell type that was present in the bronchial secretion samples was concerned, the number of macrophages was elevated in the BAL samples compared to polymorphonuclear cells regardless of the presence of VAP (* *p* < 0.05, one-way ANOVA, Figure 1C).

## 4. Discussion

MDR pathogens were involved in more than one-half of the cases of VAP in the patient population included in our study. This observation underlines the pivotal role of the ICU as a specific nosocomial environment promoting the acquisition of MDR pathogens. The clinical condition patients are in when admitted to the ICU, as estimated with the APACHE II score, can affect the induction of VAP since poor clinical condition can be a risk factor itself [7] or could be an indicator of suppressed immune system function [23]. Furthermore, even though all the patients who presented with VAP had confirmed microbial infection in the respiratory system, since some patients had coexistent sources of sepsis, we cannot exclude the possibility that part of the immunological phenomena reflected in BALF may represent a cross-talk between the lung and other systems/organs which were coinfected and have altered the immunological response of the lungs. Additionally, the fact that patients with higher scores developed VAP could be partially attributed to their already impaired overall health. Furthermore, the differences observed in the PO_2_/FIO_2_ ratio between the VAP and non-VAP patients demonstrate the role of this ratio as a parameter of respiratory distress and as a risk factor for ICU infections [26].

Low serum albumin levels were previously studied as one of the predictors of mortality in ICU patients [27,28]. Pinheiro et al. found that hypoalbuminemia is an independent predictor of mortality specifically in ICU patients with a *Pseudomonas* infection [29]. However, the role of serum albumin in predicting either the occurrence of VAP or the mortality rates in ICU patients with VAP is still unclear. Our findings in this study could contribute to the potential role of serum albumin in predicting the microbial source of VAP infection. On the other hand, the fact that the levels of total protein in the bronchial secretions from the BAL samples were elevated in the patients who developed VAP but did not have any differences regarding the type of VAP manifestation, namely with or without MDR bacteria, shows that the protein content of the bronchial lining does not follow the pattern observed in serum. This observation could be attributed to the fact that the protein content in bronchial secretions represents a local source rather than a systemic one that can be evaluated in serum samples. However, previous studies showed that an elevation in protein levels in intubated patients is indicative of airway inflammation, possibly due to intubation itself [19].

We observed increased cell counts and immune cell numbers, in particular, in the bronchial secretions from the patients diagnosed with VAP. These observations are in agreement with previous findings that highlight the impairment of the homeostasis of the lung immune system, as well as the reprogramming of lung immunity to pathogens in patients suffering from VAP [30,31]. As far as the cellular profile is concerned, inflammation due to IL-1β release appeared to be elevated in the patients with non-MDR-VAP compared to the MDR-VAP patients, probably due to the fact that the non-MDR patients had a more active immune system capable of recruiting immune cells. Different immune cell populations represent different responses. Furthermore, the immunological cell profile is crucial because immune cells produce cytokines. Increased levels of cytokines in VAP patients, especially of IL-1β, have been associated with VAP diagnosis, as well as with the differential production of early-onset cytokines, rather than cytokines such as TNF-α that are present in more advanced stages of inflammation [32]. However, the differences observed between IL-1β and TNF-α may exist because these two cytokines are involved in various signaling pathways related to VAP manifestation that can interact in many ways [33]. Furthermore, the fact that the IL-1β levels were elevated in the BAL samples and the TNF-α levels were elevated in the AC samples can be an indication of different cytokine presence in different parts of the respiratory system. In addition, endogenous inhibition of cytokines has been proposed to occur in bronchial secretions of VAP patients as well, which can alter the levels of these factors in the lung [33]. Even though an extended panel of cytokines has been proposed to participate in sepsis, IL-1β and TNF-α appear to be representative of the two basic mechanisms, namely inhibition of pathogens and tissue damage minimization [34]. The cytokine “storm”, which is expressed differently in individuals due mainly to genetic variations, consists of both pro- and anti-inflammatory cytokines. For example, interleukin (IL)-1, IL-6, IL-12, interferon (IFN)-γ, macrophage migration inhibitory factor (MIF), IL-10, transforming growth factor (TGF)-β, and IL-4 attempt to restore the immunological equilibrium and balance the pro- and anti-inflammatory cascades during sepsis [34]. Additionally, even though TNF-α and IL-1β are well-known biomarkers for sepsis and VAP, previous studies did not evaluate their values at baseline, meaning the first day of intubation, but instead after a few days under mechanical ventilation. However, our study shows that the inflammatory response is initiated from the first hours of intubation and can be used as a predictive marker of VAP manifestation.

Overall, observations regarding the levels of the biomarkers evaluated, which are present in different sampling methods, namely BAL or AC, show that both techniques are valuable for the assessment of the lung environment and function as far as inflammation is concerned. However, the observed patterns could be indicative of BAL being a better method for cellular and protein content evaluation, while AC is preferable for the evaluation of specific cytokines. Additionally, the immunological profile of the VAP patients shows a profile where the non-MDR-VAP patients reacted better to the imposed stimuli, namely infection, while the MDR-VAP patients appeared to release fewer cytokines and immune cell numbers.

The study had limitations, mainly regarding the sample size, which was relatively small to draw a definitive conclusion on the diagnostic utility of baseline BALF TNF-α and IL-1 levels to identify patients at risk for VAP. Nevertheless, this was an observational study, and we did not intend to document the precise diagnostic validity of these markers but to identify potential differences in the baseline levels in VAP and non-VAP patients. Considering the technical obstacles in recruiting patients with BALF available, the findings provide useful information that could serve as a basis for a future larger, possibly multicenter study, which could also assess genetic patterns associated with VAP manifestation and thus account for variability in clinical practices and patient demographics. Furthermore, a longitudinal assessment could provide further information regarding the effects of VAP manifestation on the quality of life of MV patients. In addition, VAP management is challenging because it incorporates various clinical practices and interventions [35]. This variability may have an impact on VAP development and outcomes. In our study, VAP management was based on recommendations. We assume that since the present study was a single-center study, variability in management may have been low; the outcomes in the VAP patients were similar to different studies conducted at our center [36]. We certainly acknowledge that at the same time, the findings of our single-center study do not permit generalization to other settings.

## 5. Conclusions

Even though the number of patients included in this study is regarded as a limitation, an overview of factors that can be used as predictive biomarkers since they are affected by susceptibility to both VAP and the presence of MDR pathogens is available. Such factors include the total protein and cytokine levels, as well as the cell count and profile in bronchial secretions obtained from mechanically ventilated patients within the first 24 h of intubation. Furthermore, a pattern of increased immune response in non-MDR infections could be attributed to a healthier immune system, since most non-MDR-VAP patients have more effective immune systems and fewer days of hospitalization by the time they are admitted to the ICU. A combination of clinical and molecular data can help attending clinicians evaluate the possibility of VAP induction in ICU-treated patients and, therefore, be prepared to apply the most suitable therapeutic approach.

## Figures and Tables

**Table 1:** Demographic and clinical characteristics of the patients enrolled in the study. Note: * *p* < 0.05, ** *p* < 0.01, Mann–Whitney test in the non-VAP (N = 16) and VAP patients (N = 13). VAP = ventilator-associated pneumonia, APACHE = Acute Physiology and Chronic Health Evaluation, SOFA = Sequential Organ Failure Assessment, NORADREN = noradrenaline, WBC = white blood cells, CRP = C-reactive protein, VT = tidal volume.

	VAP Mean (SD)	Non-VAP Mean (SD)	*p*-Value
**Age**	65.38 (4.55)	66.5 (3.83)	0.85
**APACHE II**	22.46 (2.19)	16.06 (2.05)	0.04 *
**SOFA**	9.4 (0.63)	8 (1.26)	0.48
**PO_2_/FIO_2_**	180.5 (24.6)	274.4 (26.51)	0.01 **
**PCO_2_ (cm H_2_O)**	35.77 (2.83)	33.63 (1.31)	0.47
**NORADREN (mg/mL)**	2.39 (0.22)	1.53 (0.17)	0.24
**WBC (cells × 10^3^/mL)**	13,785 (2667)	13,588 (1472)	0.94
**CRP (mg/dL)**	15.71 (2.6)	9.28 (2.29)	0.07
**Albumin (g/dL)**	2.335 (0.28)	2.67 (0.21)	0.12
**Proteins (g/kg)**	4.7 (0.34)	4.8 (0.38)	0.95
**P plateau (cm H_2_O)**	24.33 (2.3)	18.06 (0.95)	0.007 **
**VT (mL)**	464.2 (14.54)	492.5 (11.87)	0.22

**Table 2:** Demographic and clinical characteristics of the VAP patients enrolled in the study. Note: * *p* < 0.05, Mann–Whitney test in the MDR-VAP (N = 6) compared to non-MDR-VAP (N = 7) patients.

	MDR Mean (SD)	Non-MDR Mean (SD)	*p*-Value
**Age**	71.3 (7.4)	59.2 (7.59)	0.12
**APACHE II**	22 (4.76)	21.2 (0.86)	0.93
**SOFA**	9.3 (0.61)	8.25 (1.37)	0.62
**PO_2_/FIO_2_**	283.4 (29.96)	153.6 (30.87)	0.03 *
**PCO_2_ (cm H_2_O)**	31.33 (4)	42.6 (3.98)	0.12
**NORADREN (mg/mL)**	2.4 (1.15)	1.34 (0.32)	0.92
**WBC (cells × 10^3^/mL)**	14,950 (5054)	9620 (1188)	0.66
**CRP (mg/dL)**	18.08 (4.18)	10.42 (2.41)	0.18
**Albumin (g/dL)**	1.89 (0.15)	2.89 (0.66)	0.03 *
**Proteins (g/Kg)**	4.53 (0.47)	5 (0.75)	0.79
**P plateau (cm H_2_O)**	23 (3.3)	24.8 (2.9)	0.33
**VT (mL)**	482 (19.85)	432 (23.11)	0.16
**PEEP (cm H_2_O)**	7.18 (1.33)	7.57 (1.13)	0.56
**Driving pressure (cm H_2_O)**	17 (6.63)	17.57 (5.5)	0.86

**Table 3:** Infection characteristics of the patients enrolled in the study. Note: * *p* < 0.05, Mann–Whitney test in the MDR-VAP (N = 7) compared to non-MDR-VAP (N = 6) patients.

MDR	Microorganism	Coexistent Sepsis Source	Day of VAP Manifestation	Total MV Duration
1	*Klebsiella pneumoniae*	Blood	15	20
2	*Acinetobacter baumanii*	Blood	15	19
3	*Pseudomonas aeruginosa*	Lung	7	7
4	*Pseudomonas aeruginosa*	Abdomen	7	9
5	*Stenotrophomonas maltophila*	Lung	3	7
6	*Pseudomonas aeruginosa*	Pleura	4	9
7	*Klebsiella pneumoniae*	Lung	4	10
Total = 7			Mean: 7.85 * *p* < 0.05 (*p* = 0.0476) compared to the non-MDR microorganisms	Mean: 11.57 * *p* < 0.05 (*p* = 0.0344) compared to the non-MDR microorganisms
**Non-MDR**				
1	MSSA	Aorta	2	2
2	*Candida* spp.	Lung	3	5
3	*Klebsiella pneumoniae*	Lung	4	5
4	*Candida* spp.	Lung	5	9
5	*Pseudomonas aeruginosa*	Lung	7	10
6	*Klebsiella pneumoniae*	Lung	15	15
Total = 6			Mean: 6	Mean: 7.66

**Table 4:** Percentage of antibiotic resistance in the bacteria identified.

MDR Pathogens		Non-MDR Pathogens	
Antimicrobial	%	Antimicrobial	%
Meropenem	71.43	Meropenem	50
Piperacillin/tazobactam	85.71	Piperacillin/tazobactam	66.67
Gentamicin	57.14	Gentamicin	16.67
Colistin	57.14	Colistin	33.33
Ampicillin	100	Ampicillin	83.33
Ampicillin/sulbactam	85.71	Ampicillin/sulbactam	66.67
Ciprofloxacin	100	Ciprofloxacin	83.33
Tigecycline	71.43	Tigecycline	33.33

**Table 5:** Total protein, IL-1β, and TNF-α levels in the BAL and AC samples from the MV patients. Note: * *p* < 0.05, ** *p* < 0.01, *** *p* < 0.001, **** *p* < 0.0001, one-way ANOVA in the non-VAP (N = 16) compared to VAP patients (N = 13) and in MDR-VAP (N = 7) compared to non-MDR-VAP (N = 6).

	Non-VAP		VAP		VAP-MDR		VAP-Non MDR	
	BAL	AC	BAL	AC	BAL	AC	BAL	AC
Total protein (μg/mL)	8.94 * (*p* = 0.0468) Compared to BAL VAP	12.04	15.13	15.18	18.02	14.64	12.83	15.83
IL-1β (ng/mL)	752.5 ** (*p* = 0.0012) Compared to BAL VAP	427.5 * (*p* = 0.0277) Compared to BAL non VAP	962.5	661.5 **** (*p* < 0.0001) Compared to BAL VAP	1528	715.8 * (*p* = 0.036) Compared to BAL VAP-MDR	1083 ** (*p* = 0.0082) Compared to BAL VAP-MDR	710.0 *** (*p* = 0.0002) Compared to BAL VAP-non MDR
TNFα (ng/mL)	738.3	1135 * (*p* = 0.0205) Compared to BAL non VAP	986.0 *** (*p* = 0.0004) Compared to AC VAP	1601 ** (*p* = 0.0067) Compared to AC non VAP	832.0	1621 ** (*p* = 0.0095) Compared to BAL VAP-MDR	1060	1450 **** (*p* < 0.0001) Compared to BAL VAP-non MDR

## Data Availability

The original contributions presented in this study are included in the article. Further inquiries can be directed at the corresponding author.
